# Adversity in Infancy and Childhood Cognitive Development: Evidence From Four Developing Countries

**DOI:** 10.3389/ijph.2022.1604503

**Published:** 2022-12-13

**Authors:** W. Samuel Manalew, Vidhura S. Tennekoon, Jusung Lee, Bethesda O’Connell, Megan Quinn

**Affiliations:** ^1^ East Tennessee State University, Johnson City, TN, United States; ^2^ Indiana University Purdue University Indianapolis, Indianapolis, IN, United States; ^3^ University of Texas at San Antonio, San Antonio, TX, United States

**Keywords:** developing countries, childhood, adverse experiences, infancy, cognitive development, peabody picture vocabulary test, young lives study

## Abstract

**Objectives:** We investigated whether adverse experiences at age 1 (AE-1) affect the level of and change in cognition during childhood using harmonized data from four developing countries.

**Methods:** Data included children born in 2001/2002 and were followed longitudinally in 2006/2007 and in 2009/2010 by Young Lives study in Ethiopia, India, Peru, and Vietnam. Childhood cognition was measured using the Peabody Picture Vocabulary Test (PPVT) at ages 5 (PPVT-5) and 8 (PPVT-8). We also examined the effect on a change in cognition between age 5–8 (PPVT-Change). The AE-1 scores were constructed using survey responses at age 1. The ordinary least squares regression was used for estimation.

**Results:** We found that children with higher adversities as infants had lower cognition scores at ages 5 and 8. The change in cognition between the two ages was also generally smaller for those with severe adversities at infancy. The negative association between adversities and childhood cognition was strongest for India.

**Conclusion:** The results provide policy relevant information for mitigation of undesirable consequences of early life adversities through timely interventions.

## Introduction

As people all over the world are experiencing a sustained long period of adverse experiences which keeps their level of stress high, researchers are overwhelmingly focusing on mental health consequences of the pandemic [1–3]. A particularly vulnerable group to various form of adversities are children due to their limited understanding of an adverse event, abrupt withdrawal from school, social life and outdoor activities, social isolation and separation from caregivers due to quarantine policies, increased domestic violence, changes in diets and dietary patterns, among other reasons [4–7]. While the short-term psychological and psychiatric repercussions of these adverse conditions on children have been widely discussed, the long-term consequences are to be known only after several years. Early-life experiences, both positive and adverse, lay foundations for health and wellbeing in later life, making early-life experiences an important public health issue [8–11]. Children exposed to various forms of adversities early in life are at increased risk for a broad range of developmental disorders including delayed brain (cognitive) development [12] and poor mental health [13]. They also face an increased risk of morbidity as adults and early mortality [14]. Children who experience adversities before the age of 3 are more vulnerable to the effects of adversities compared to children who experience the adversities at older ages [13].

Actions to prevent and reduce the negative consequences of early-life adversities must be the obligation of every society and is in accordance with the Sustainable Development Goals of the United Nations [15, 16]. And it is important that these actions are based on scientific evidence. Most past research focused on investigating the effect of early-life adversities on adult outcomes, and relatively fewer studies examined the link between early-life adversities and childhood outcomes. However, for policy purposes, studying the effects of early-life adversities on childhood outcomes is preferred over studying their effects on adult outcomes because childhood outcomes manifest early in life and, therefore, are more amenable to policy interventions [17]. Moreover, much of the evidence on the impact of early adversities on outcomes later in life is based on data from the developed countries, and there is a dearth of scientific evidence in the context of low and middle income countries (LMICs) [14]. The current context sheds light on why the findings based on developed counties could not be generalized to LMICs. While most types of adversities faced by a child living in a developed country are also shared by children in LMICs, their burden of economic shocks such as the loss of family income and food insecurity arising from crop damage and loss of livestock are not common in the developed world. In addition, a development disorder of a child living in a LMIC is less likely to be diagnosed and treated than a disorder of a child living in a developed county and therefore the negative consequences of an adverse event during the early childhood on cognitive development are more likely to long prevail.

This study, using multi-country longitudinal data from four LMICs (Ethiopia, India, Peru, and Vietnam), examined whether exposure to adverse experiences during the first year of life (age 1) is associated with early-childhood (age 5) and mid-childhood (age 8) cognition, measured by the Peabody Picture Vocabulary Test (PPVT). In addition, the study examined whether the change in cognition between age 5 and age 8, measured using the unpredicted change in PPVT scores between those ages, is associated with adversities in infancy. Understanding these associations is essential because childhood cognitive development is a strong predictor of adulthood cognition and human capital [8]. Our measure of adversity is broad-based and covers a variety of adverse events relevant in a LMIC context including economic, environmental, emotional and health shocks. Our findings shed light on an important international public health issue; how adverse effects of early childhood experiences propagate over time retarding the cognitive development of children in developing country settings. These research findings can be helpful in formulating public health policies which would mitigate long-term risks of adverse conditions many children in LMICs are currently facing through cost-effective policy interventions.

## Methods

### The Data and the Study Population

The current study used data from the Young Lives (YL) study, conducted in four LMICs: Ethiopia, India, Peru, and Vietnam. The YL is a multi-country longitudinal sample of 2,000 children in each of the four countries who were aged 6–18 months in 2002, the young cohort. Data were also collected in each country from 1000 children aged 7–8 years in 2002, the old cohort, and follow-ups were conducted in 2006 (round 2) and 2009 (round 3). In this study, we used data from approximately 8000 children from the younger cohort, who were aged 1 year in round 1 and 5 years and 8 years in rounds 2 and 3, respectively. The YL data includes detailed information on health, nutrition, adverse household shocks, cognitive development, education, as well as the socioeconomic and demographic status of the household. An important advantage of the YL is its low attrition rate. We used deidentified, publicly available data for analysis from the UK Data Archive [18].

### Variables

**Childhood cognitive development:** cognition was measured by the Peabody Picture Vocabulary Test (PPVT), administered to participants both at age 5 (round 2), and age 8 (round 3). The PPVT is a norm-referenced test used to measure receptive vocabulary and to assess verbal ability [19, 20] PPVT test score is positively correlated with other common measures of intelligence, such as the Wechsler and McCarthy Scales [21]. The PPVT was originally developed in the English language in 1959, has been updated several times, and developed in many other languages. This YL study used version III of the test [19]. The PPVT test was administered individually, orally, and untimed, under the condition that the test taker is shown a series of sheets, each with four pictures displayed, and the participant selects the picture that best represents a target word presented orally by the examiner [22]. Each child was given a version of the test in a language they are most comfortable, and the fieldworkers were instructed not to administer the test if an appropriate language version of the test was not available. In Peru, India, and Vietnam the test was administered in Spanish, Telugu and Vietnamese, respectively. In Ethiopia, the test was administered in Amharic, Tigray or Oromo. The PPVT scores were reconstructed by YL such that cognitive scores are comparable across rounds and age cohorts [22] but the reconstructed scores of different language versions of the test are still not comparable. To facilitate comparison of the magnitude of the effect of the adversities on cognition across different languages, and thus across the four countries, we standardized the PPVT scores within the languages in which the tests were administered by converting to Z-scores. The standardization of the data also controls for the differences based on how the data were managed in different countries. We used three outcomes in our analysis: standardized PPVT score at age 5 (PPVT-5), standardized PPVT score at age 8 (PPVT-8), and the unpredicted change in PPVT score between age 5 and age 8 (PPVT-Change). The standardized PPTV scores measure the receptive vocabulary relative to the average level of children in the particular language group.

In deriving the PPTV-Change: 1) a linear regression was performed, with PPVT-8 as the dependent variable and PPVT-5 and the other covariates as independent variables; 2) PPVT-8 score was predicted from the regression; and 3) the standardized PPVT-Change score was calculated as the actual PPVT-8 minus the predicted PPVT-8 score, divided by the standard error of the estimate from the regression equation. Thus, the standardized PPVT-Change score provides a measure of what the observed level of cognition at age 8 was in comparison to what would be predicted based on cognition at age 5, i.e., the unpredicted change. The change score derived from this standardized regression-based method has been shown to be an accurate and reliable method for capturing both normal cognitive change and diagnostic change [23, 24].

**Adverse experiences at age 1 (AE-1):** the children’s exposure to various adversities at age 1 was assessed based on responses by parents or caregivers to a range of survey questions about the children’s early-life experiences. Based on the responses, we defined eight types of adverse experiences at age 1: poor health (two questions), parental divorce or separation (1 question), incarcerated household member (1 question), family’s exposure to adverse economic shocks (three questions), households’ exposure to adverse natural and environmental events (two questions), family’s exposure to crime (3 questions), and parental neglect (one question). This broad range of adverse events captures different types of adverse shocks potentially affecting the mental health of a child in a LMIC setting.

All of the above individual adversities were combined to create an aggregate measure of adversity in infancy, the AE-1 score. For each question with a dichotomous response, a child was assigned a score of “1” if a parent or a caregiver indicated exposure of the child to an adverse condition in question, the child was given “0”, otherwise. For the question with multiple response options, child’s weight category, a score was assigned such that “0” represents the lowest level of adversity (not underweight), a score of “1” represents moderate adversity (moderately underweight), and a score of “2” represents severe adversity (severely underweight). The AE-1 score was constructed as the sum of the above individual category scores, which ranged from 0 to 18, with higher scores indicating worse early-life experiences. After constructing the AE-1 score, the final measure was derived by grouping the observations into 4 categories based on the severity of adverse early experiences at infancy: None (AE-1 = 0), Mild (AE-1 = 1), Moderate (AE-1 = 2 or 3) and High (AE-1 = 4 or more). Table 1 presents the survey questions used to define the AE-1 score. It is possible that the relationship between the adverse experiences and the childhood outcomes is confounded by endogenous variables included in the creation of the aggregate adversity index. As a robustness check, an alternative AE-1 score was constructed the same way but excluding the potentially endogenous adverse events noted in Table 1.

**TABLE 1:** Questions used to derive the Adverse Experiences at age 1 (AE-1) score; Adversity in infancy and childhood cognitive development in four developing countries (Ethiopia, India, Peru, and Vietnam—2002–2009).

Questions	Responses	Score value
1. Poor child health (0–3)		
Q1. Child weight for age^a^	Not underweight	0
	Moderately underweight	1
	Severely underweight	2
Q2. Child had serious injury	No	0
	Yes	1
2. Parental separation or divorce (0–1)		
Q1. Shock-divorce or separation happen^a^	No	0
	Yes	1
3. Incarcerated household member (0–1)		
Q1. Household member imprisoned^a^	No	0
	Yes	1
4. Economic Shocks (0–3)		
Q1. Shock-decrease in food availability	No	0
	Yes	1
Q2. Shock-death of livestock	No	0
	Yes	1
Q3. Household experienced loss of source of income	No	0
	Yes	1
5. Natural and environmental shocks (0–1)		
Q1. Shock-crop failure	No	0
	Yes	1
Q2. Shock-natural disaster	No	0
	Yes	1
6. Quality of care (0–1)		
Q1. Caregiver’s relationship to YL child^a^	Biological Parent	0
	Other	1
7. Crimes (0–3)		
Q1. Shock theft of crops	No	0
	Yes	1
Q2. Shock theft of livestock	No	0
	Yes	1
Q3. Household victim of any crime?	No	0
	Yes	1
8. Other family shocks (0–4)		
Q1. Shock-sever illness or injury of family member	No	0
	Yes	1
Q2. Unplanned birth^a^	No	0
	Yes	1
Q3. Death in the family	No	0
	Yes	1
Q4. Family displacement/migration	No	0
	Yes	1

^a^ The alternative index constructed without potentially endogenous adverse events excludes these events.

**Covariates:** In our regression models, we controlled for confounding variables at individual, household, community, as well as social and contextual levels that could influence childhood cognitive development. These included age, gender, area of residence (rural/urban), maternal education, religion, and household wealth. The YL wealth index is a primary measure of the socio-economic status of households within the YL sample. It was constructed from three indices: housing quality, access to services, and ownership of consumer durable household items [25]. The average YL wealth index produces values between 0 and 1, with a higher wealth index indicating better socio-economic status [25]. We also included country and community dummy variables in the regression model to control for country and community level time-invariant characteristics, such as overall economic, social and educational development, and other contexts of the individual communities or countries the YL sample was selected from. All the confounding variables in our baseline model were drawn from the round 1 survey in 2002, when the average age of the children was 1 year. Descriptive statistics of the variables used are presented in Table 2.

**TABLE 2:** Characteristics of the sample of children participating in the Young Lives study; Adversity in infancy and childhood cognitive development in four developing countries (Ethiopia, India, Peru, and Vietnam—2002–2009).

	Total sample, N = 8,062, %	Ethiopia (*n* = 1,999), %	India (*n* = 2,011) %	Peru (*n* = 2,052) %	Vietnam (*n* = 2,000) %
A. Categorical Variables					
AE-1 Score Category					
None (0)	34.5	16.1	32.0	41.6	48.0
Mild (1)	25.6	14.7	23.1	36.1	28.0
Moderate (2–3)	25.3	31.5	28.1	20.8	21.1
High (4+)	14.7	37.7	16.8	1.6	3.0
Sex					
Male	52.0	52.5	53.8	50.1	51.4
Female	48.0	48.0	46.0	50.0	49.0
Mother’s Education					
None	40.6	59.9	62.0	12.0	27.9
Primary	34.7	31.9	19.8	45.3	42.2
Secondary and above	25.0	8.0	18.0	42.7	30.0
Religion					
Non-Christian	54.0	17.2	95.1	5.7	97.8
Christian	46.0	83.0	5.0	94.0	2.0
Residence					
Urban	37.4	35.0	25.3	68.5	20.0
Rural	62.6	65.0	74.7	31.5	80.0
Wealth Quantiles					
Q1	25.0	25.0	25.0	25.1	25.1
Q2	25.0	25.2	25.0	25.0	25.0
Q3	25.0	24.8	25.0	25.1	26.3
Q4	25.0	24.9	25.0	24.9	23.7
B. Continuous Variables					
Variable					
Age of the child	11.7 (3.5)	11.7 (3.6)	11.8 (3.5)	11.5 (3.5)	11.6 (3.2)
Family size	5.4 (2.2)	5.7 (2.2)	5.4 (2.3)	5.7 (2.3)	4.9 (1.8)
PPVT_5_raw score	28.6 (18.5)	21.4 (12.4)	27.4 (21.1)	29.2 (17.8)	37.0 (18.2)
PPVT_8_raw score	72.6 (35.0)	79.2 (44.2)	58.5 (30.4)	58.9 (17.6)	94.0 (28.6)

Data are mean (standard deviation) for continuous variables and percentages for categorical variables. AE_1 indicates Adverse Experiences at age 1.

### Analysis

The Ordinary Least Square (OLS) regression model was fitted to assess the association between the AE-1 score and the PPVT-5, PPVT-8, and PPVT-Change. To examine whether the associations between AE-1 and the PPVT scores were confounded by adversities later in life, and thus to check the robustness of our results, we conducted a sensitivity analysis in which we control for adversities experiences at age 5 (AE-5) in addition to AE-1.

The estimation was based on the following specification:

$$Y_{ijsc}=\beta_{0}+\beta_{1}AEI_{ijsc}+\beta_{2}C_{ijsc}+\beta_{3}H_{jsc}+\theta_{sc}+r_{c}+\mu_{ijsc}$$ (1)

where each observation is for individual child *i* in household *j* in sentinel (community/cluster of villages) *s, and country c*. The dependent variable $Y_{ijs}$ denotes the PPVT-5, PPVT-8, or PPVT-Change score, $AEI_{ijsc}$ is a categorical variable ranging from 0 to 5, with higher values indicating more severe adversities. $C_{ijsc}$ denotes child characteristics, $H_{jsc}$ denotes household characteristics, $\theta_{sc}$ is sentinel/community fixed effects, $r_{c}$ is a country fixed effect, and $\mu_{ijsc}$ are the idiosyncratic error terms. Standard errors were clustered at the child level to account for the fact that the same child was included in the model twice (at age 1 and at age 5 or 8).

The main analysis was first conducted for the pooled data sample, harmonized data from all four countries, and then repeated separately for each country to explore any country-specific differences. The raw PPVT scores were used for the individual country analysis. Standardizing the PPVT variables was not needed for the individual country analysis because PPVT scores from different languages were analyzed separately, and comparability was not an issue. Data management and subsequent analyses were conducted using Stata software, version 15 [26].

## Results

The pooled data from all countries resulted in a sample of 8,062 children, approximately 2,000 children from each country. After dropping children with missing observations in any of the explanatory variables used in our main analysis, a total sample of 7,327 children was included in the pooled model. Over one third (34.5%) of the children had an AE-1 score of zero, with the remaining 65.0% having AE-1 scores of 1 or more. About half of the children (48.0%) are *female* and the remaining 52.0% are male. Over one-third of the children (40.6%) had a mother with no education, and nearly two-third (62. 6%) lived in rural areas.

There were significant variations across nations over most variables. Ethiopia and India have lower proportions (16.1% and 32.0%) of children with AE-1 score of “zero”, compared with 41.6% and 48.0% for Peru and Vietnam, respectively. Conversely, smaller proportions of children in Peru and Vietnam experienced severe adversities, compared to those in Ethiopia and India. The raw PPVT scores also vary, with PPVT-5 scores ranging from 21.4 in Ethiopia to 37.0 in Vietnam, and PPVT-8 scores ranging from 58 in India to 94 in Vietnam. However, as discussed in *Variables* section , our standardization approach eliminates these differences and makes the PPTV scores comparable across counties. Cross-contextual and socioeconomic differences were also seen in socioeconomic covariates, including maternal education, rural/urban status of residence, and wealth. Minor differences were noted in the average family size and average age of children (Table 2).

The association between adverse experiences in infancy and childhood cognitive development is shown in Table 3. We find that children with adverse experiences in infancy achieved lower test scores in childhood compared with children who had no adverse experiences, the reference group. We also find an inverse relationship between the test scores and the level of adversity. Compared with children with no adversity in infancy, those with mild, moderate, and high adversities as infants had lower PPVT-5 scores, with estimated coefficients of −0.06, −0.09, and −0.09, respectively. Children with “High” adversity level as infants had a lower PPVT-8 and PPVT-Change scores, with estimated coefficients of −0.15 and −0.16, respectively. The alternative measure without potentially endogenous components produces comparable results (Table 3). Results from the measure with only the endogenous variables are given in the Supplementary Material.

**TABLE 3:** Associations between adverse experiences at age 1 and childhood cognitive development among children in Young Lives study; Adversity in infancy and childhood cognitive development in four developing countries (Ethiopia, India, Peru, and Vietnam—2002–2009).

	Adversity score based on all variables^a^			Adversity score based on exogeneous variables^b^		
	PPVT score at age 5	PPVT score at age 8	Change in PPVT score	PPVT score at age 5	PPVT score at age 8	Change in PPVT score
AE_1 Category						
None	Ref.	Ref	Ref.	Ref.	Ref.	Ref.
Mild (1)	−0.06**	−0.01	−0.02	−0.04	−0.02	−0.04
	[−0.12, −0.00]	[−0.07, 0.05]	[−0.09, 0.05]	[−0.10, 0.01]	[−0.07, 0.04]	[−0.10, 0.03]
Moderate (2–3)	−0.09***	0.01	0.02	−0.09***	−0.02	0.01
	[−0.15, −0.03]	[−0.05, 0.07]	[−0.05, 0.10]	[−0.15, −0.04]	[−0.08, 0.04]	[−0.06, 0.09]
High (4+)	−0.09**	−0.15***	−0.16***	−0.01	−0.12**	−0.14**
	[−0.17, −0.01]	[−0.23, −0.07]	[−0.25, −0.07]	[−0.10, 0.08]	[−0.22, −0.03]	[−0.25, −0.03]
Age of the child	0.05***	0.04***	0.01***	0.05***	0.04***	0.01***
	[0.04, 0.06]	[0.04, 0.05]	[0.00, 0.02]	[0.04, 0.06]	[0.04, 0.05]	[0.00, 0.02]
Female	−0.06**	−0.08***	0.00	−0.05**	−0.07***	0.00
	[−0.10, −0.01]	[−0.12, −0.03]	[−0.06, 0.05]	[−0.10, −0.01]	[−0.12, −0.03]	[−0.05, 0.05]
Family size	−0.01	−0.01	0.00	−0.01	−0.01*	0.00
	[−0.02, 0.00]	[−0.02, 0.00]	[−0.01, 0.01]	[−0.02, 0.00]	[−0.02, 0.00]	[−0.01, 0.01]
Mother Education						
None	Ref.	Ref	Ref.	[0.00, 0.00]	[0.00, 0.00]	[0.00, 0.00]
Primary	0.17***	0.17***	0.03	0.17***	0.17***	0.04
	[0.11, 0.23]	[0.11, 0.23]	[−0.04, 0.11]	[0.11, 0.23]	[0.11, 0.24]	[−0.04, 0.11]
Secondary	0.54***	0.48***	0.01	0.54***	0.48***	0.01
	[0.46, 0.62]	[0.40, 0.56]	[−0.09, 0.11]	[0.46, 0.62]	[0.40, 0.56]	[−0.08, 0.11]
Religion						
Non-Christian	Ref.	Ref.	Ref.	Ref.	Ref.	Ref.
Christian	0.12**	−0.04	0.01	0.12**	−0.04	0.01
	[0.02, 0.22]	[−0.14, 0.06]	[−0.12, 0.13]	[0.02, 0.22]	[−0.15, 0.06]	[−0.12, 0.13]
Residence						
Urban	Ref.	Ref.	Ref.	Ref.	Ref.	Ref.
Rural	−0.28***	−0.19***	0.13***	−0.28***	−0.20***	0.12**
	[−0.36, −0.20]	[−0.27, −0.11]	[0.04, 0.22]	[−0.36, −0.21]	[−0.28, −0.12]	[0.03, 0.21]
Wealth Quantiles						
Q1	Ref.	Ref	Ref.	Ref.	Ref.	Ref.
Q2	0.09***	0.22***	0.17***	0.09***	0.23***	0.17***
	[0.03, 0.15]	[0.16, 0.29]	[0.09, 0.24]	[0.03, 0.15]	[0.16, 0.29]	[0.10, 0.25]
Q3	0.19***	0.42***	0.24***	0.20***	0.42***	0.24***
	[0.12, 0.26]	[0.34, 0.49]	[0.15, 0.33]	[0.12, 0.27]	[0.35, 0.50]	[0.15, 0.33]
Q4	0.57***	0.62***	0.23***	0.58***	0.62***	0.24***
	[0.48, 0.66]	[0.53, 0.71]	[0.13, 0.34]	[0.48, 0.67]	[0.53, 0.72]	[0.13, 0.35]
Country Fixed Effects						
Ethiopia	Ref	Ref	Ref	Ref.	Ref.	Ref.
India	0.08	−0.12**	−0.07	0.08	−0.11*	−0.05
	[−0.03, 0.19]	[−0.24, −0.01]	[−0.20, 0.07]	[−0.03, 0.20]	[−0.23, 0.00]	[−0.19, 0.09]
Peru	−0.49***	−0.44***	−0.03	−0.48***	−0.42***	0.00
	[−0.37, −0.13]	[−0.52, −0.37]	[−0.12, 0.06]	[−0.56, −0.40]	[−0.50, −0.35]	[−0.09, 0.08]
Vietnam	−0.25***	−0.40***	−0.14*	−0.24***	−0.38***	−0.12
	[−0.97, −0.58]	[−0.53, −0.27]	[−0.29, 0.01]	[−0.36, −0.12]	[−0.51, −0.26]	[−0.27, 0.04]
Total	6021	6078	5681	6021	6078	5681

***p < 0.001; **p < 0.005; and *p < 0.01, AE_1 indicates Adverse Experiences at age 1, PPVT, peabody picture vocabulary test.

^a^ All variables in Table 1 were used in the calculation of the aggregate adversity score.

^b^ Potentially endogenous variables from Table 1 were excluded from the calculation of the aggregate adversity score.

Results from the analysis that controls for adversities both at age 1 and age 5 is given in Table 4 Results from this analysis showed similar effects, with higher adversity scores in the first year of life associated with lower scores in cognitive tests during childhood. Compared with children with no adversity in infancy, those with mild, moderate, and high adversities as infants had lower PPVT-5 scores, with estimated coefficients of −0.06, −0.08, and −0.08, respectively. Children with the “High” adversity level as infants had a lower PPVT-8 and PPVT-Change scores, with estimated coefficients of −0.13 and −0.14, respectively. Again, the alternative measure without potentially endogenous components produces comparable results (Table 4). Results from the measure with only the endogenous variables are given in the Supplementary Material.

**TABLE 4:** Associations between adverse experiences at age 1 and childhood cognitive development among children in Young Lives study-a sensitivity analysis; Adversity in infancy and childhood cognitive development in four developing countries (Ethiopia, India, Peru, and Vietnam—2002–2009).

	Adversity score based on all variables^a^			Adversity score based on exogenous variables^b^		
	PPVT score at age 5	PPVT score at age 8	Change in PPVT score^c^	PPVT score at age 5	PPVT score at age 8	Change in PPVT score^c^
AE-1 score category						
None (0)	Ref.	Ref.	Ref.	Ref	Ref	Ref
Mild (1)	−0.06*	0.00	−0.02	−0.04	−0.02	−0.03
	[−0.11, 0.00]	[−0.06, 0.05]	[−0.09, 0.05]	[−0.10, 0.01]	[−0.07, 0.04]	[−0.10, 0.03]
Moderate (2, 3)	−0.08***	0.02	0.03	−0.10***	−0.01	0.02
	[−0.15, −0.02]	[−0.04, 0.08]	[−0.04, 0.10]	[−0.15, −0.04]	[−0.08, 0.05]	[−0.05, 0.09]
High (4+)	−0.08**	−0.13***	−0.14***	−0.01	−0.11**	−0.13**
	[−0.16, −0.00]	[−0.21, −0.05]	[−0.24, −0.05]	[−0.10, 0.08]	[−0.21, −0.02]	[−0.24, −0.02]
AE-5 score category						
None (0)	Ref.	Ref.		Ref.	Ref.	
Mild (1)	−0.05*	0.00		0.05*	0.02	
	[−0.10, 0.01]	[−0.05, 0.06]		[−0.00, 0.10]	[−0.03, 0.07]	
Moderate (2, 3)	−0.03	−0.04		−0.01	−0.11***	
	[−0.10, 0.04]	[−0.11, 0.03]		[−0.09, 0.06]	[−0.19, −0.03]	
High (4+)	−0.07*	−0.12***		0.00	−0.29***	
	[−0.14, 0.01]	[−0.20, −0.04]		[−0.08, 0.09]	[−0.43, −0.15]	
Age of the child	0.05***	0.04***	0.01***	0.05***	0.04***	0.01***
	[0.04, 0.06]	[0.04, 0.05]	[0.00, 0.02]	[0.04, 0.06]	[0.04, 0.05]	[0.00, 0.02]
Female	−0.06**	−0.08***	0.00	−0.05**	−0.08***	0.00
	[−0.10, −0.01]	[−0.12, −0.03]	[−0.06, 0.05]	[−0.10, −0.01]	[−0.12, −0.03]	[−0.05, 0.05]
Family size	−0.01	−0.01	0.00	−0.01	−0.01	0.00
	[−0.02, 0.00]	[−0.02, 0.00]	[−0.01, 0.01]	[−0.02, 0.00]	[−0.02, 0.00]	[−0.01, 0.01]
Mother Education						
None	Ref.	Ref.	Ref.	Ref	Ref	Ref
Primary	0.17***	0.17***	0.03	0.17***	0.17***	0.04
	[0.11, 0.23]	[0.11, 0.23]	[−0.04, 0.11]	[0.11, 0.23]	[0.11, 0.23]	[−0.04, 0.11]
Secondary or higher	0.54***	0.48***	0.01	0.54***	0.48***	0.01
	[0.46, 0.62]	[0.40, 0.56]	[−0.09, 0.11]	[0.46, 0.62]	[0.40, 0.56]	[−0.08, 0.11]
Christian	0.12**	−0.05	0.01	0.12**	−0.05	0.01
	[0.02, 0.22]	[−0.15, 0.05]	[−0.12, 0.13]	[0.02, 0.22]	[−0.15, 0.05]	[−0.12, 0.13]
Rural	−0.28***	−0.18***	0.12***	−0.29***	−0.19***	0.12**
	[−0.35, −0.20]	[−0.26, −0.10]	[0.04, 0.22]	[−0.36, −0.21]	[−0.27, −0.11]	[0.03, 0.21]
Wealth Quantiles						
Q1	Ref.	Ref.	Ref.	Ref	Ref	Ref
Q2	0.09***	0.22***	0.17***	0.09***	0.23***	0.17***
	[0.03, 0.15]	[0.15, 0.28]	[0.09, 0.24]	[0.03, 0.15]	[0.16, 0.29]	[0.10, 0.25]
Q3	0.19***	0.41***	0.23***	0.20***	0.42***	0.24***
	[0.11, 0.26]	[0.33, 0.49]	[0.14, 0.32]	[0.12, 0.27]	[0.34, 0.49]	[0.15, 0.33]
Q4	0.56***	0.61***	0.23***	0.58***	0.62***	0.24***
	[0.47, 0.66]	[0.52, 0.70]	[0.13, 0.34]	[0.49, 0.67]	[0.53, 0.71]	[0.13, 0.34]
Country fixed effects						
Ethiopia	Ref.	Ref.	Ref.	Ref	Ref	Ref
India	0.08	−0.13***	−0.06	0.09	−0.12**	−0.05
	[−0.04, −0.19]	[−0.25, −0.02]	[−0.20, 0.08]	[−0.03,0.20]	[−0.24, −0.01]	[−0.19, 0.09]
Peru	−0.50***	−0.46***	−0.02	−0.48***	−0.43***	0
	[−0.58, −0.42]	[−0.54, −0.38]	[−0.11, 0.07]	[−0.56, −0.41]	[−0.51, −0.36]	[−0.09, 0.09]
Vietnam	−0.26***	−0.69***	−0.13*	−0.23***	−0.39***	−0.11
	[−0.38, −0.14]	[−0.88, −0.50]	[−0.28, 0.02]	[−0.35, −0.11]	[−0.52, −0.27]	[−0.26, 0.04]
Total	6021	6078	5681	6021	6078	5681

***p < 0.001; **p < 0.005; and *p < 0.01, AE_1 indicates Adverse Experiences at age 1, PPVT, peabody picture vocabulary test.

^a^ All variables in Table 1 were used in the calculation of the aggregate adversity score.

^b^ Potentially endogenous variables from Table 1 were excluded from the calculation of the aggregate adversity score.

^c^ AE-5 was excluded because it is controlled in the preliminary regression used to estimate the predicted value of PPTV-8.

The significant effects of the early life adversities on childhood cognition were also seen in country-stratified models for India and Vietnam, but not for the other countries. Both PPVT-5 and PPVT-8 had a statistically significant negative associations with adversities in infancy for India. Adversity in infancy was statistically significantly associated with only PPVT-5 for Vietnam. When the alternative adversity measure without the endogenous components was used, results for Ethiopia at age 8 turned significant while the results for India turned insignificant. The associations for Vietnam remained significant (Table 5). Results from the measure with only the endogenous variables are given in the Supplementary Material.

**TABLE 5:** Associations between adverse experiences in infancy and childhood cognitive development among children in Young Lives study by individual country; Adversity in infancy and childhood cognitive development in four developing countries (Ethiopia, India, Peru, and Vietnam—2002–2009).

	Adversity score based on all variables^a^			Adversity score based on exogenous variables^b^		
	PPVT score at age 5	PPVT score at age 8	Change in PPVT Score	PPVT score at age 5	PPVT score at age 8	Change in PPVT Score
Ethiopia						
AE_1 score categories^c^						
None (0)	Ref.	Ref.	Ref.	Ref.	Ref.	Ref.
Moderate (1)	−1.71	−5.2	−0.12	−0.15	−5.59	−0.16
	[−5.20, 1.78]	[13.97, 3.57]	[−0.38, 0.13]	[−2.65, 2.35]	[−12.43, 1.24]	[−0.36, 0.04]
High (2+)	−0.91	−5.56	−0.17	−0.15	−6.34*	−0.20**
	[−3.48, 1.67]	[12.91, 1.78]	[−0.38, 0.05]	[−2.01, 1.72]	[−12.76, 0.07]	[−0.39, −0.01]
India						
AE_1 score category						
None (0)	Ref.	Ref.	Ref.	Ref.	Ref.	Ref.
Moderate (1)	−1.55	0.01	0.02	−1.06	−1.17	0.00
	[−3.88, 0.77]	[−3.54, 3.56]	[−0.12, 0.15]	[−3.42, 1.30]	[−4.60, 2.26]	[−0.13, 0.12]
High (2+)	−3.79***	−2.80*	−0.07	−1.44	−0.99	−0.01
	[−5.92, −1.66]	[−6.12, 0.52]	[−0.19, 0.06]	[−3.47, 0.59]	[−4.41, 2.43]	[−0.14, 0.12]
Peru						
AE_1 score category						
None (0)	Ref.	Ref.	Ref.	Ref.	Ref.	Ref.
Moderate (1)	−0.07	0.02	−0.01	0.21	0.27	0.00
	[−1.35, 1.20]	[−1.43, 1.48]	[−0.12, 0.09]	[−1.00, 1.42]	[−1.13, 1.67]	[−0.10, 0.11]
High (2+)	0.01	0.03	0.00	0.17	1.41	0.10
	[−1.51, 1.53]	[−1.74, 1.79]	[−0.14, 0.13]	[−1.65, 1.98]	[−0.60, 3.43]	[−0.05, 0.25]
Vietnam						
AE_1 score category						
None (0)	Ref.	Ref.	Ref.	Ref.	Ref.	Ref.
Moderate (1)	−1.93**	0.09	0.01	−2.54***	−0.80	−0.04
	[3.50, −0.37]	[−2.44, 2.62]	[−0.09, 0.11]	[−4.08, −1.00]	[−3.32, 1.73]	[−0.14, 0.06]
High (2+)	−3.37***	−0.39	0.01	−2.52***	−1.19	−0.01
	[5.09, −1.64]	[−3.11, 2.34]	[−0.10, 0.11]	[−4.39, −0.65]	[−4.33, 1.95]	[−0.14, 0.11]

Results were adjusted for age, sex, family size, maternal education, urban/rural residence, religion, and wealth, ***p < 0.001; **p < 0.005; and *p < 0.01, AE_1 indicates Adverse Experiences at age 1, PPVT, peabody picture vocabulary Test.

^a^ All variables in Table 1 were used in the calculation of the aggregate adversity score.

^b^ Potentially endogenous variables from Table 1 were excluded from the calculation of the aggregate adversity score.

## Discussion

This study examined the association between adversities in early infancy and childhood cognitive development using a harmonized multi-country dataset. We found a statistically significant association between the adverse experiences at age 1 and the level of cognitive development at ages 5 and 8 measured using standardized PPTV scores, as well as the cognitive development between the two ages. Children who faced adversities as infants had lower PPVT scores during childhood, with PPVT scores getting worse as the severity of the adversities increased. Severe adversity in infancy was also associated with change in cognition between early and middle childhoods. The association remains robust after controlling adversities later in childhood. These results suggest that early adversities have long-lasting effects on childhood cognitive development.

The association of high AE-1 scores with the unpredicted change in PPTV scores in between the ages of 5 and 8 is particularly interesting and policy relevant. This model investigates how early adversities affect cognitive development during later years, net of the same effect through contemporaneous adverse experiences. The children who face adversities during infancy are vulnerable to similar events in their later life and the association between PPTV and AE-1 scores may show this effect rather than the effect of AE-1 score itself. The difference between the actual PPTV score at 8 and the predicted scores using all available information at age 5 including the AE-5 score rules out this possibility. Similarly, the results of our robustness checks using on PPTV-5 and PPTV-8 after controlling for adversities faced at age 5, rules out the possibility of this effect. Another potential limitation is the endogeneity bias in parameter estimates due to some of the constituting components of the AE-1 index [27]. To circumvent the issue, we estimated all our models using an alternative measure which excludes those components. The results rule out any significant bias.

While the main findings of this study are applicable to all the countries, some important differences across countries should be highlighted. In Ethiopia, the association between AE-1 and the PPVT scores was negative but not statistically significant. The association was negative and statistically significant for India. In Peru, the association was statistically significant for PPVT-8 and PPVT-Change, while it was statistically significant only for PPVT-5 in Vietnam. The results show that the negative effects of the adversities in the first year of life on childhood cognitive development were weaker in Ethiopia, stronger in India, strengthened over time in Peru, and weakened over time in Vietnam. This could be due to contextual or policy related differences across the countries towards addressing various adversities children experience in their early lives. While the overall findings provide evidence for the effects of early-life adversities, countries would need to assess their unique cultural or social conditions in interpreting the findings and implementing effective policy interventions [28]. One previous study finds an association between environmental shocks and higher PPVT scores of children in Ethiopia and Peru. The results for India and Vietnam, however, are either insignificant or in the opposite direction [29].

Although the primary objective of the study was to examine associations between the AE-1 and the PPVT scores, it is also noteworthy to reflect on the socioeconomic and demographic factors impacting childhood cognitive development. Female children scored less in the PPVT test at both age 5 and age 8. Residing in rural areas was also negatively associated with the PPVT scores. Children who live in households with large family sizes scored less in the cognition tests. Similarly, children of less-educated mothers scored less in the PPVT tests. These findings are largely consistent with what has been reported in the literature, suggesting that these factors should be seriously considered in efforts to reduce the negative impacts of early-life adversity experiences on childhood cognitive development [30, 31].

This study is not without limitations. First, in the study, we constructed measures for AE-1 scores based on items available from the survey. Even though our AE-1 score was based on an extensive set of variables, these factors were by no means exhaustive. However, we believe that they provide considerable information for understanding the early-life experiences of children given wide dimensions of questions assessed, such as health, poverty, neglect, environments, and items relevant to measure various forms of adversities a child could experience at age 1. Not every aspect of childhood cognition is captured by the PPVT test as it mainly measures verbal cognition. However, measures of cognitive ability such as PPVT are preferred measures of educational attainment to measures such as years of schooling because cognitive skills are a stronger determinant of adulthood outcomes than school attainment [32]. Even though we controlled for a range of confounding factors, some unobservable factors could still confound the relationship between AE-1 and cognitive development. For example, the unobservable factors, such as household characteristics, institutional help during crisis, urban/rural residence, and caregiver’s education, could explain some of the differences in the PPVT test scores [33]. Finally, cohorts in the YL survey may not be nationally representative as the survey participants were recruited from certain communities in the countries. The sample from India, for example, is unlikely to capture the heterogeneity across states, because the YL children from India came from 20 communities in two of the Indian states (states of Andhra Pradesh and Telangana) [22]. Further, although YL study has taken measures to address limitations in the study design, still there may concerns around the adaptation of measures in cross-cultural settings, translation, and cross-cultural equivalence [20].

Despite the limitations, the data from these four countries provide critical findings for policy implications, especially in developing countries, where many infants are exposed to various forms of adversities. Given that adverse experiences in early life are disproportionately high in developing countries, estimating its negative effects on childhood cognitive development will inform policymakers to design interventions that can reverse the negative effects of early adversities. Since the topic has been understudied in developing country settings, findings in this study may be novel in those contexts. Our findings suggest that early interventions to improve neonatal conditions through policy interventions could result in better childhood outcomes, which will have practical implications for improved adulthood outcomes, too. Additionally, policymakers are advised to seriously consider parental education and remedial education policies that could reverse the adverse effects of poor neonatal conditions.

Early life cognition is an essential component of health and wellbeing across a person’s life course. Our findings suggest that policies that aim to successfully manage a society’s future could do so by effectively addressing early life adversities.

## Data Availability

Publicly available datasets were analyzed in this study. This data can be found here: www.younglives.org.uk *.* A data use agreement was made with the University of Essex and the right to use the collections provided *via* the UK Data Service and the UK Data Archive was granted.
