# Supplementary material for: Adversity in Infancy and Childhood Cognitive Development: Evidence From Four Developing Countries
Source: Int J Public Health. 2022 Dec 13;67:1604503. doi: 10.3389/ijph.2022.1604503 (PMC9792379; doi:10.3389/ijph.2022.1604503)
Supplement: Supplementary file 1 [file Table1.DOCX]

**Journal:** International Journal of Public Health

**Title:** Adversity in infancy and childhood cognitive development: evidence from four developing countries

**Supplementary material**

**Table S1** Characteristics of the sample of children participating in the Young Lives study; Adversity in infancy and childhood cognitive development in four developing countries (2002-2009)

|  | Total Sample  N=8,062  % | Ethiopia  (n=1,999)  % | India  (n=2,011)  % | Peru  (n=2,052)  % | Vietnam  (n=2,000)  % |
| --- | --- | --- | --- | --- | --- |
| **A. Categorical Variables** |  |  |  |  |  |
| **AE-1 Score Category ^a^** |  |  |  |  |  |
| None (0) | 45.3 | 32.9 | 45.8 | 50.3 | 59.3 |
| Mild (1) | 25.7 | 24.2 | 20.6 | 36.6 | 25.9 |
| Moderate (2-3) | 21.8 | 42.9 | 33.7 | 13.1 | 14.9 |
| High (4+) | 7.2 | -- | -- | -- | -- |
| **AE_1 Score Category ^b^** |  |  |  |  |  |
| None (0) | 67.7 | 50.2 | 60.9 | 81.4 | 77.4 |
| Mild (1) | 20.5 | 23.5 | 26.3 | 15.5 | 17.3 |
| Moderate (2-3) | 11.7 | 26.3 | 12.9 | 3.12 | 5.4 |
| **Sex** |  |  |  |  |  |
| Male | 52.0 | 52.5 | 53.8 | 50.1 | 51.4 |
| Female | 48.0 | 48.0 | 46.0 | 50.0 | 49.0 |
| **Mother's Education** |  |  |  |  |  |
| None | 40.6 | 59.9 | 62.0 | 12.0 | 27.9 |
| Primary | 34.7 | 31.9 | 19.8 | 45.3 | 42.2 |
| Secondary and above | 25.0 | 8.0 | 18.0 | 42.7 | 30.0 |
| **Religion** |  |  |  |  |  |
| Non-Christian | 54.0 | 17.2 | 95.1 | 5.7 | 97.8 |
| Christian | 46.0 | 83.0 | 5.0 | 94.0 | 2.0 |
| **Residence** |  |  |  |  |  |
| Urban | 37.4 | 35.0 | 25.3 | 68.5 | 20.0 |
| Rural | 62.6 | 65.0 | 74.7 | 31.5 | 80.0 |
| **Wealth Quantiles** |  |  |  |  |  |
| Q1 | 25.0 | 25.0 | 25.0 | 25.1 | 25.1 |
| Q2 | 25.0 | 25.2 | 25.0 | 25.0 | 25.0 |
| Q3 | 25.0 | 24.8 | 25.0 | 25.1 | 26.3 |
| Q4 | 25.0 | 24.9 | 25.0 | 24.9 | 23.7 |
| **B. Continuous Variables** |  |  |  |  |  |
| Variable |  |  |  |  |  |
| Age of the child | 11.7 (3.5) | 11.7 (3.6) | 11.8 (3.5) | 11.5 (3.5) | 11.6 (3.2) |
| Family size | 5.4 (2.2) | 5.7 (2.2) | 5.4 (2.3) | 5.7 (2.3) | 4.9 (1.8) |
| PPVT_5_raw score | 28.6 (18.5) | 21.4 (12.4) | 27.4 (21.1) | 29.2 (17.8) | 37.0 (18.2) |
| PPVT_8_raw score | 72.6 (35.0) | 79.2 (44.2) | 58.5 (30.4) | 58.9 (17.6) | 94.0 (28.6) |

Data are mean (standard deviation) for continuous variables and percentages for categorical variables. AE_1 indicates Adverse Experiences at age 1

a Adversity scores based on c exogeneous variables

b Adversity scores based on endogenous variables

**Table S2** Associations between adverse experiences at age 1 and childhood cognitive development among children in Young Lives study; Adversity in infancy and childhood cognitive development in four developing countries (2002-2009)

|  | **AIE index based on endogenous variables ^a^** | | |
| --- | --- | --- | --- |
|  | PPVT  score  at age 5 | PPVT  score  at age 8 | Change in  PPVT  score |
| **AIE_1 Category** |  |  |  |
| None | Ref. | Ref. | Ref. |
| Mild (1) | -0.09*** | -0.05* | -0.05 |
|  | [-0.15, -0.04] | [-0.11,0.00] | [-0.11,0.02] |
| High (2+) | -0.09** | -0.12*** | -0.12** |
|  | [-0.17, -0.02] | [-0.19, -0.04] | [-0.21, -0.03] |
| Age of the child | 0.05*** | 0.04*** | 0.01*** |
|  | [0.04,0.06] | [0.04,0.05] | [0.00,0.02] |
| Female | -0.06** | -0.08*** | -0.01 |
|  | [-0.10, -0.01] | [-0.12, -0.04] | [-0.06,0.04] |
| Family size | -0.01 | -0.01* | 0.00 |
|  | [-0.02,0.00] | [-0.02,0.00] | [-0.01,0.01] |
| **Mother Education** |  |  |  |
| None | Ref. | Ref. | Ref. |
| Primary | 0.17*** | 0.17*** | 0.03 |
|  | [0.11,0.23] | [0.11,0.23] | [-0.04,0.11] |
| Secondary | 0.54*** | 0.48*** | 0.01 |
|  | [0.46,0.62] | [0.40,0.56] | [-0.09,0.11] |
| **Religion** |  |  |  |
| Non-Christian | Ref. | Ref. | Ref. |
| Christian | 0.12** | -0.04 | 0.01 |
|  | [0.03,0.22] | [-0.14,0.07] | [-0.11,0.13] |
| **Residence** |  |  |  |
| Urban | Ref. | Ref. | Ref. |
| Rural | -0.29*** | -0.20*** | 0.12** |
|  | [-0.37, -0.21] | [-0.28, -0.12] | [0.03,0.21] |
| **Wealth Quantiles** |  |  |  |
| Q1 | Ref. | Ref. | Ref. |
| Q2 | 0.08*** | 0.23*** | 0.17*** |
|  | [0.02,0.15] | [0.16,0.29] | [0.10,0.25] |
| Q3 | 0.19*** | 0.42*** | 0.24*** |
|  | [0.12,0.26] | [0.35,0.50] | [0.15,0.33] |
| Q4 | 0.57*** | 0.62*** | 0.24*** |
|  | [0.48,0.66] | [0.53,0.72] | [0.13,0.35] |
| **Country Fixed Effects** |  |  |  |
| Ethiopia | Ref. | Ref. | Ref. |
| India | 0.09 | -0.10* | -0.05 |
|  | [-0.02,0.20] | [-0.22,0.01] | [-0.18,0.09] |
| Peru | -0.50*** | -0.43*** | -0.02 |
|  | [-0.57, -0.42] | [-0.51, -0.35] | [-0.10,0.07] |
| Vietnam | -0.24*** | -0.38*** | -0.12 |
|  | [-0.36, -0.12] | [-0.50, -0.25] | [-0.27,0.03] |
| Total | 6021 | 6078 | 5681 |

***p<0.001; **p<0.005; and *p<0.01, AE_1 indicates Adverse Experiences at age 1, PPVT, Peabody Picture Vocabulary Test

a Potentially endogenous variables used in the calculation of the aggregate adversity score

**Table S3** Associations between adverse experiences at age 1 and childhood cognitive development among children in Young Lives study-a sensitivity analysis; Adversity in infancy and childhood cognitive development in four developing countries (2002-2009)

|  | AIE index based on endogenous variables ^a^ | | |
| --- | --- | --- | --- |
|  | PPVT  score  at age 5 | PPVT  score  at age 8 | Change in  PPVT  score |
| **AE-1 score category** |  |  |  |
| None (0) | Ref | Ref | Ref |
| Mild (1) | -0.09*** | -0.05* | 0 |
|  | [-0.15, -0.04] | [-0.11,0.00] | [-0.07,0.07] |
| High (2+) | -0.09** | -0.12*** | 0 |
|  | [-0.17, -0.02] | [-0.19, -0.04] | [-0.09,0.09] |
| **AE-5 score category ^b^** |  |  |  |
| None (0) | Ref | Ref |  |
| Mild (1) | 0.00 | 0.00 |  |
|  | [0.00,0.00] | [0.00,0.00] |  |
| High (2+) | 0.00 | 0.00 |  |
|  | [0.00,0.00] | [0.00,0.00] |  |
| Age of the child | 0.05*** | 0.04*** | 0.01*** |
|  | [0.04,0.06] | [0.04,0.05] | [0.00,0.02] |
| Female | -0.06** | -0.08*** | 0.00 |
|  | [-0.10, -0.01] | [-0.12, -0.04] | [-0.05,0.05] |
| Family size | -0.01 | -0.01* | 0.00 |
|  | [-0.02,0.00] | [-0.02,0.00] | [-0.01,0.01] |
| **Mother Education** |  |  |  |
| None | Ref | Ref | Ref |
| Primary | 0.17*** | 0.17*** | 0.04 |
|  | [0.11,0.23] | [0.11,0.23] | [-0.04,0.11] |
| Secondary or higher | 0.54*** | 0.48*** | 0.02 |
|  | [0.46,0.62] | [0.40,0.56] | [-0.08,0.11] |
| Christian | 0.12** | -0.04 | 0.01 |
|  | [0.03,0.22] | [-0.14,0.07] | [-0.12,0.13] |
| Rural | -0.29*** | -0.20*** | 0.11** |
|  | [-0.37, -0.21] | [-0.28, -0.12] | [0.02,0.20] |
| **Wealth Quantiles** |  |  |  |
| Q1 | Ref | Ref | Ref |
| Q2 | 0.08*** | 0.23*** | 0.18*** |
|  | [0.02,0.15] | [0.16,0.29] | [0.10,0.25] |
| Q3 | 0.19*** | 0.42*** | 0.24*** |
|  | [0.12,0.26] | [0.35,0.50] | [0.16,0.33] |
| Q4 | 0.57*** | 0.62*** | 0.24*** |
|  | [0.48,0.66] | [0.53,0.72] | [0.14,0.35] |
| **Country fixed effects** |  |  |  |
| Ethiopia | Ref | Ref | Ref |
| India | 0.09 | -0.10* | -0.04 |
|  | [-0.02,0.20] | [-0.22,0.01] | [-0.17,0.10] |
| Peru | -0.50*** | -0.43*** | 0.01 |
|  | [-0.57, -0.42] | [-0.51, -0.35] | [-0.08,0.10] |
| Vietnam | -0.24*** | -0.38*** | -0.09 |
|  | [-0.36, -0.12] | [-0.50, -0.25] | [-0.24,0.05] |
| N | 6021 | 6078 | 5681 |

***p<0.001; **p<0.005; and *p<0.01, AE_1 indicates Adverse Experiences at age 1, PPVT, Peabody Picture Vocabulary Test

a Potentially endogenous variables used in the calculation of the aggregate adversity score

^b^ AE-5 was excluded because it’s controlled in the preliminary regression used to estimate the predicted value of PPTV-8.

**Table S4** Associations between adverse experiences in infancy and childhood cognitive development among children in Young Lives study by individual country; Adversity in infancy and childhood cognitive development in four developing countries (2002-2009)

|  | Adversity index based on endogenous variables a | | |
| --- | --- | --- | --- |
|  | PPVT score  at age 5 | PPVT score  at age 8 | Change in  PPVT Score |
|  | **Ethiopia** | | |
| **AEI_1 score categories ^c^** |  |  |  |
| None (0) | Ref. | Ref. | Ref. |
| Moderate (1) | -1.42 | -4.29 | -0.12 |
|  | [-3.55,0.70] | [-10.88,2.31] | [-0.31,0.07] |
| High (2+) | -1.00 | -0.44 | -0.01 |
|  | [-3.35,1.35] | [-7.38,6.50] | [-0.21,0.19] |
|  | **India** | | |
| None (0) | Ref. | Ref. | Ref. |
| Moderate (1) | -3.56*** | -3.12** | -0.08 |
|  | [-5.42, -1.70] | [-6.05, -0.18] | [-0.19,0.03] |
| High (2+) | -6.14*** | -9.13*** | -0.31*** |
|  | [-8.54, -3.73] | [-12.58, -5.68] | [-0.44, -0.18] |
|  | **Peru** | | |
| **AEI_1 score category** |  |  |  |
| None (0) | Ref. | Ref. | Ref. |
| Moderate (1) | -0.22 | -0.86 | -0.05 |
|  | [-1.85,1.41] | [-2.67,0.94] | [-0.19,0.08] |
| High (2+) | -0.63 | -6.72** | -0.56*** |
|  | [-4.11,2.84] | [-11.95, -1.49] | [-0.96, -0.15] |
|  | **Vietnam** | | |
| **AEI_1 score category** |  |  |  |
| None (0) | Ref. | Ref. | Ref. |
| Moderate (1) | -3.34*** | -0.09 | 0.03 |
|  | [-4.89, -1.79] | [-3.03,2.84] | [-0.09,0.14] |
| High (2+) | -1.48 | -0.03 | 0.04 |
|  | [-4.96,1.99] | [-4.40,4.35] | [-0.12,0.20] |

Results were adjusted for age, sex, family size, maternal education, urban/rural residence, religion, and wealth, ***p<0.001; **p<0.005; and *p<0.01, AE_1 indicates Adverse Experiences at age 1, PPVT, Peabody Picture Vocabulary Test

a Potentially endogenous variables used in the calculation of the aggregate adversity score
